# Supplementary material for: A resource for development and comparison of multimodal brain 3 T MRI harmonisation approaches
Source: Imaging Neurosci (Camb). 2023 Dec 4;1:imag-1-00042. doi: 10.1162/imag_a_00042 (PMC12007558; doi:10.1162/imag_a_00042)
Supplement: Supplementary Material [file imag_a_00042-supp.pdf]

Supplementary Information for

"A resource for development and comparison of multimodal brain 3T MRI harmonisation approaches"

S. Warrington[a*], A. Ntata[a*], O. Mougin[b], J. Campbell[c], A. Torchi[a], M. Craig[a], F. Alfaro-Almagro[c], K. L. Miller[c], P. S. Morgan[a,f], M. Jenkinson[c,d,e], S. N. Sotiropoulos[a,c,f]

[a]Sir Peter Mansfield Imaging Centre, School of Medicine, University of Nottingham, UK
[b]Sir Peter Mansfield Imaging Centre, School of Physics and Astronomy, University of Nottingham, UK
[c]Wellcome Centre for Integrative Neuroimaging, FMRIB Centre, Nuffield Department of Clinical Neurosciences (NDCN), University of Oxford, UK
[d]Australian Institute for Machine Learning (AIML), School of Computer and Mathematical Sciences, The University of Adelaide, Australia
[e]South Australian Health and Medical Research Institute (SAHMRI), Adelaide, Australia
[f]National Institute for Health Research (NIHR) Nottingham Biomedical Research Centre, Nottingham, UK

*Equal contribution

Corresponding: Stamatios Sotiropoulos, Stamatios.sotiropoulos@nottingham.ac.uk

Supplementary Information

Total readout time equations

Here, we report the equations used by dcm2niix to calculate the total readout time for each vendor.

Siemens: https://github.com/rordenlab/dcm2niix/issues/130

$$EffectiveEchoSpacing = \frac{1}{BWPPPE \times MatrixSizePE}$$

$$TotalReadoutTime = EffectiveEchoSpacing \times (MatrixSizePE - 1)$$

where $BWPPPE$ is the bandwidth per pixel in the phase encoding direction and $MatrixSizePE$ is the dimension of the image reconstruction matrix in the phase encoding direction.

GE: https://github.com/rordenlab/dcm2niix/tree/master/GE

$$TotalReadoutTime = \left(ceil\left(\frac{1}{RF} \times \frac{MatrixSizePE}{AssetR}\right) * RF\right) - 1) \times ES \times 0.000001$$

where RF is a round factor applied conditionally such that RF is 2 (Full Fourier) or 4 (Partial Fourier), $MatrixSizePE$ is the image acquisition matrix dimension in the phase encoding direction, $AssetR$ is the in-plane acceleration factor.

Philips: https://osf.io/xvguw/wiki/home/

$$ActualEchoSpacing = \frac{WaterFatShift}{ImagingFrequency \times 3.4 \times (EPIFactor + 1)}$$

$$TotalReadoutTime = ActualEchoSpacing \times EPIFactor$$

$$EffectiveEchoSpacing = \frac{TotalReadoutTime}{MatrixSizePE - 1}$$

where $ActualEchoSpacing$ is the acquisition echo spacing, $EPIFactor$ is the equivalent to the echo train length plus 1, $MatrixSizePE$ is the dimension of the image reconstruction matrix in the phase encoding direction.

Note: in our data, the acquisition and reconstruction matrix dimensions are equal.

Minor subject protocol deviations

The majority of subjects were acquired using the same protocols. However, for some subjects there were minor deviations in some protocol parameters that we describe here for completeness.

GE MR 750 rfMRI: For one subject (03286), we acquired two versions of the rfMRI data with 1) an isotropic spatial resolution of 2.4mm and 2) a spatial resolution of 2.2x2.2x3.4mm. Neither matched the 3.3mm isotropic data acquired for other subjects. As such, we excluded this subject's rfMRI data from the analyses. For 6 subjects (03286, 03997, 10975, 12813, 14482, 14221) there was a mismatch in PE direction between dMRI and fMRI that we accounted for in the processing pipeline.

Philips Achieva dMRI: In most cases, the dMRI protocol included 6 b=0 s/mm^2 volumes. Four subjects (13305, 13192, 14229, 14230) were acquired with 2 b=0 s/mm^2 volumes.

Philips Ingenia dMRI: In most cases, dMRI data were acquired using an in-plane acceleration factor of 1.5 (TE=98ms, TR=4.4s). For 4 subjects (13305, 13192, 14229, 14230), the dMRI data were acquired using an in-plane acceleration factor of 2 (TE=92ms, TR=3.9s).

Scanner software details

Software version was maintained for the majority of sessions. Siemens Prisma scanners used software version Syngo MR E11 and the Siemens Trio scanner used Syngo B17. The GE M750 used software version DV 24.0. For the Philips scanners, the software version was upgraded during the study. For the Achieva, 6 subjects (03286, 03997, 13192, 13305, 14229, 14230) were acquired with version 5.3.0.3 and the remaining 4 subjects (10975, 12813, 14221, 14482) were acquired with version 5.6.1.1. For the Ingenia, 6 subjects (03286, 03997, 13192, 13305, 14229, 14230) were acquired with version 5.3.1.0 and the remaining 4 subjects (10975, 12813, 14221, 14482) were acquired with version 5.6.1.0.

Effect of subject age and inter-scan delays

As noted, due to national lockdowns during the Covid-19 pandemic, our data includes longer than ideal delays between within-subject repeat scans. There is potential for these delays to affect our data in meaningful ways. To assess the effect of subject age and scan delays on the variability of IDPs across scanners and subjects, we regressed out these two variables (i.e. age and scan delays) from the IDPs and using these residualised IDPs we reproduced the relative difference between between-scanner and between-subject variability (bottom plot of Figure 6 from the main text). To do so, for each vector of IDPs Y (N sessions long), we estimated a vector of regressors $\tilde{\beta} = pinv(A) \cdot Y$, where the A is an N by M array containing two demeaned regressors (subject age and scan delays) for each session. The regressed IDPs were then calculated as $Y_r = Y - A \cdot \tilde{\beta}$. Next, we calculated the relative difference between the between-scanner and between-subject variabilities using Y (as in Figure 6) and using the IDPs Y_r, where the effect of age and scan delay has been regressed out. Results are shown in Supplementary Figure 10, where we find no widespread differences between the original and regressed measures, with some small differences for certain IDP groups such as the atlas-based dMRI FA measures.

Supplementary Tables

Subject ID	Sex	Age	Within-scan repeats	Between-scan interval (days)	Within-scan interval (days)
03286	M	48	No	206	N/A
03997	M	37	No	261	N/A
10975	M	25	No	434	N/A
12813	F	24	No	562	N/A
13192	M	47	Yes	314	38 (Prisma 32)
13305	M	42	No	671	N/A
14221	M	25	No	555	N/A
14229	M	35	Yes	298	205 (Prisma 64)
14230	F	25	Yes	203	92 (Trio)
14482	M	24	Yes	500	378 (Achieva)
Summary:	80% M	Mean = 33.2<br>Std = 9.9	Total = 4	Mean = 432<br>Std = 153	Mean = 150<br>Std = 160

Supplementary Table 1 *– Subject demographics and time taken to acquire all scans.*

dMRI	ES (ms)	MB	PI	PF	IF (MHz)	WFS (pix)	BW (Hz/px)	Matrix size PE	EES (ms)	TRT (ms)
**Philips Achieva**	0.67	None	2	80%	127.8	17.0	25.5	112	0.35	39
**Philips Ingenia**	0.67	3	1.5	80%	127.8	32.7	18.1	112	0.67	74
**GE MR750**	0.68	None	2	None	127.8	-	-	104	0.34	35
**Siemens Prisma 32ch**	0.67	3	None	75%	123.2	-	14.4	104	0.67	69
**Siemens Prisma 64ch**	0.67	3	None	75%	123.2	-	14.4	104	0.64	69
**Siemens Trio**	0.69	3	None	75%	123.2	-	13.9	104	0.69	71

Supplementary Table 2 *– The effective echo spacing and total readout times used in distortion correction processing for dMRI data. Values displayed are as reported from dcm2niix (v1.0.20211006) and as used in the equations presented in the Supplementary Information. Where available we report all relevant parameters across vendors. Unavailable parameters are denoted by '-'. We report both the water fat shift and bandwidth for Philips: water fat shift is used in dcm2niix to calculate the effective echo spacing and we report bandwidth to allow comparison across scanners. ES = echo spacing; MB = multiband factor; PI = in-plane acceleration factor; PF = partial Fourier; IF = imaging frequency; WFS = water fat shift (pixels); BW = phase encoding bandwidth per pixel (Hz/pixel); Matrix size PE = image (acquisition and reconstruction) matrix size in the phase encoding direction; EES = effective echo spacing; TRT = total readout time.*

rfMRI	ES (ms)	MB	PI	PF	IF (MHz)	WFS (pix)	BW (Hz/px)	Matrix size PE	EES (ms)	TRT (ms)
**Philips Achieva**	0.61	4	None	80%	127.8	25.4	17.1	96	0.61	58
**Philips Ingenia**	0.62	4	1.5	None	127.8	17.1	25.5	96	0.41	39
**GE MR750**	0.54	None	2	None	127.8	-	-	64	0.21	13
**Siemens Prisma 32ch**	0.64	8	None	None	123.2	-	17.8	88	0.64	56
**Siemens Prisma 64ch**	0.64	8	None	None	123.2	-	17.8	88	0.64	56
**Siemens Trio**	0.69	8	None	None	123.2	-	16.5	88	0.69	60

Supplementary Table 3 *– As in Supplementary Table 2 but for rfMRI data.*

QC Metric	Category	T1w	dMRI	rfMRI
Spatial Contrast to Noise Ratio (CNR)	Noise related	/		
Angular Contrast to Noise Ratio (CNR)			/	
Signal to Noise Ratio (SNR)		/	/	
Temporal SNR (tSNR)				/
Coefficient of Joint Variation (CJV)	Outlier and artefact related	/		
AFNI's Outlier index (AQI)				/
AFNI's Outlier Ratio (AOR)				/
Outliers (Intensity dropout)			/	
Quality Index 1 (QI 1)		/		
Absolute Motion	Motion related		/	/
Relative Motion			/	
Framewise Displacement (FD)				/
Eddy Current Distortions	Distortion related		/	
Susceptibility Induced Distortions			/	
Full-width-half-maximum (FWHM)	Blurring related	/		/
Entropy Focused Criterion (EFC)		/		

Supplementary Table 4 *– The image quality metrics (IQMs) used to assess anatomical (T1w), diffusion MRI and functional MRI data quality. IQMs are derived using MRIQC for T1w and fMRI data and eddyQC for dMRI data.*

IDP group		Between-scanner vs. within-scanner			Between-scanner vs. biological		
		Median	Quantile (5th)	Quantile (95th)	Median	Quantile (5th)	Quantile (95th)
T1w	Atlas-based cortical volumes	223.2	34.8	731.2	-55.1	-78.7	60.7
	Subcortical volumes (FIRST)	31.8	3.3	112.7	-69.4	-82.9	-43.6
	Tissue volumes (SIENAX)	134.8	104.6	464.8	-71.4	-89.4	-42.1
SWI	T2* subcortex	189.9	33.6	412.8	70.0	-11.1	132.1
T1w FreeSurfer	Subcortical volumes	51.3	20.2	144.3	-58.0	-83.4	-47.8
	Cortical curvature (DK)	41.6	-4.6	144.0	-53.4	-71.7	-21.1
	Cortical surface area (DK)	61.2	8.3	239.2	-81.0	-86.0	-53.0
	Cortical thickness (DK)	51.3	-0.9	173.7	-48.3	-70.7	1.5
	Cortical volume (DK)	80.8	9.9	205.0	-72.7	-81.7	-50.3
Atlas-based dMRI	FA	212.1	100.3	472.5	-11.9	-52.0	38.0
	L1	177.6	68.3	299.9	-22.2	-49.2	13.3
	L2	224.2	102.9	434.9	-7.1	-39.8	57.6
	L3	219.5	89.9	396.7	0.8	-45.3	64.7
	MD	215.1	101.8	472.9	0.2	-32.5	70.1
	MO	180.1	57.9	453.2	-30.4	-63.9	16.7
Tractography-based dMRI	FA	216.1	80.5	442.6	21.9	-34.5	83.5
	L1	133.7	48.0	336.8	-11.6	-46.8	41.1
	L2	153.8	48.8	341.6	-10.9	-39.5	42.8
	L3	174.9	53.6	321.5	-2.0	-41.2	48.2
	MD	134.3	40.2	282.9	-16.5	-48.7	16.8
	MO	129.0	44.2	226.3	-18.7	-50.4	32.7
rfMRI	d=25 connectivities	-38.1	-79.4	27.8	4.4	-79.2	215.8
	d=25 node amplitudes	10.9	-9.9	61.0	-18.6	-27.5	-1.1

Supplementary Table 5 *– The mean relative difference in IDP-group-wise coefficient of variation of between-scanner repeats relative to within-scanner repeat and biological variability. Between-scanner vs within-scanner calculated as (between - within)/within. Between-scanner vs biological calculated as (between - biological)/biological.*

Supplementary Figures

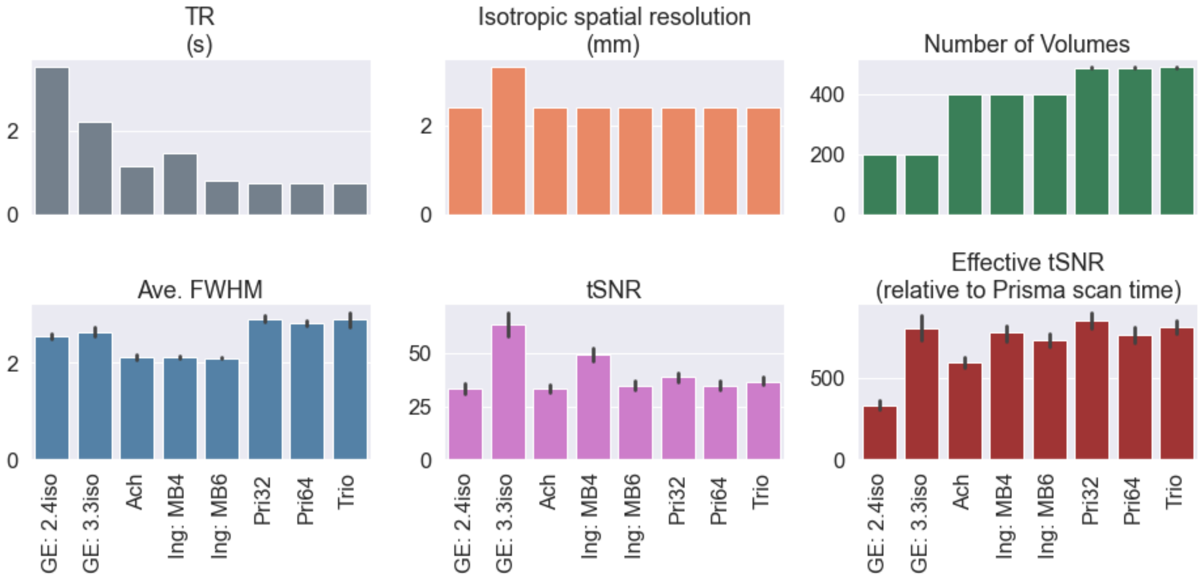

Supplementary Figure 1 *– Comparisons of image-quality metrics (IQMs) for different version of the rfMRI protocols across scanners. Each bar represents the mean (and standard deviation – error bars) of the reported IQM across subjects.*

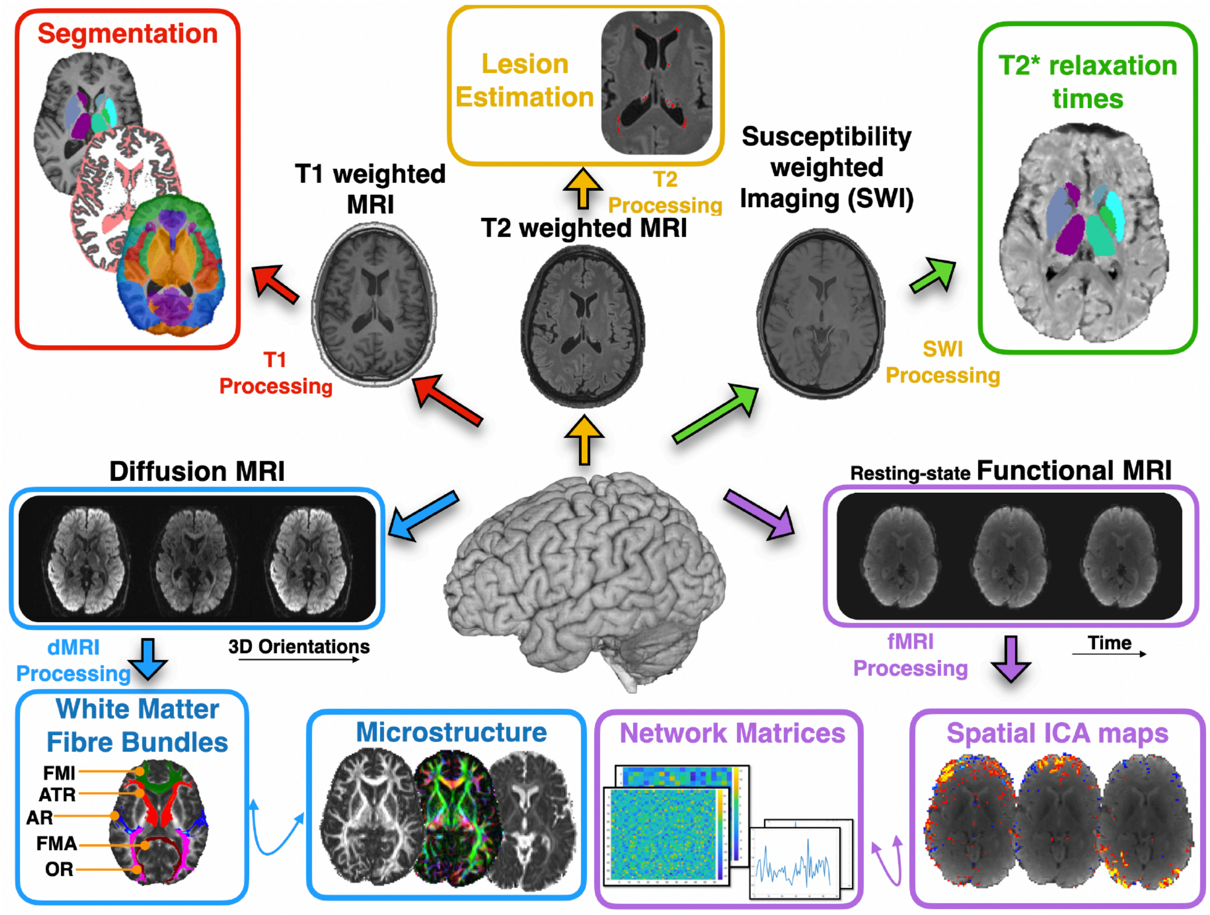

Supplementary Figure 2 – *Overview of the features extracted from each modality. The data from each modality were processed using a modified version of the UK Biobank pipeline to obtain a comprehensive set of imaging features across all scanning sessions.*

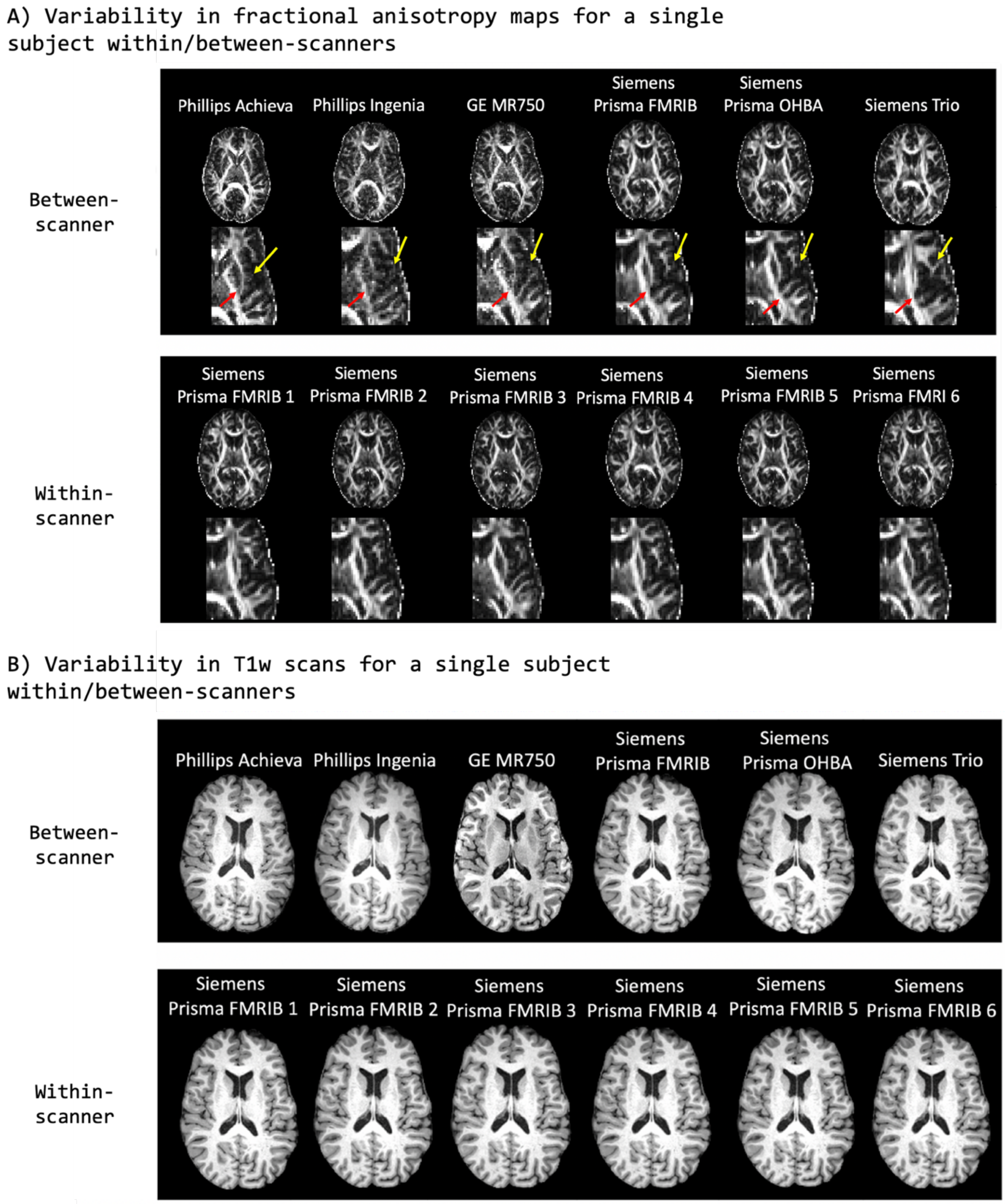

Supplementary Figure 3 *– Comparisons of modality-specific variability in quality within/between-scanners. A) Variability in fractional anisotropy (FA) maps between (top) and within (bottom) scanners. Qualitatively, between-scanner variability is greater than within-scanner variability. Specifically, grey/white matter contrast varies appreciably between scanners. Yellow arrows highlight grey matter regions with between-scanner differences. In addition, noise and spatial inhomogeneities are variable across scanners: red arrows highlight such differences. B) Variability in T1-weighted images between (top) and within (bottom) scanners. There are few noticeable qualitative differences in the data between scanners. This is comparable to within-scanner data which is similarly consistent. Note: all data are shown in their respective native spaces.*

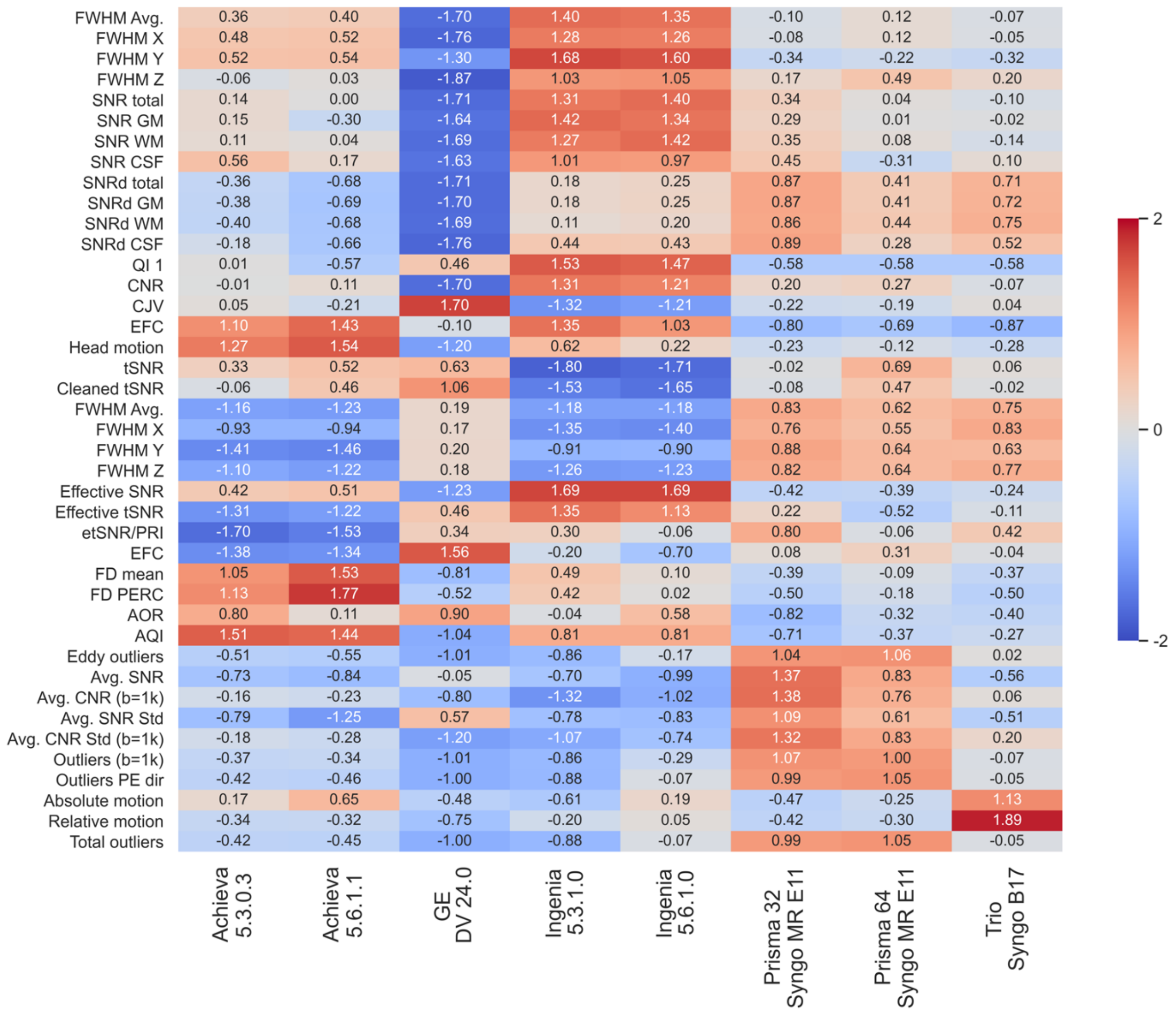

Supplementary Figure 4 *– Heatmap of Image quality metrics (IQM) variability across scanner and software version. Each quality metric for each subject was z-scored across scanners/software version. The Z-scores were then averaged across the 10 subjects. We exclude within-scanner repeats. Higher positive or negative values represent large deviations from the mean z-scored IQM across scanners/subjects.*

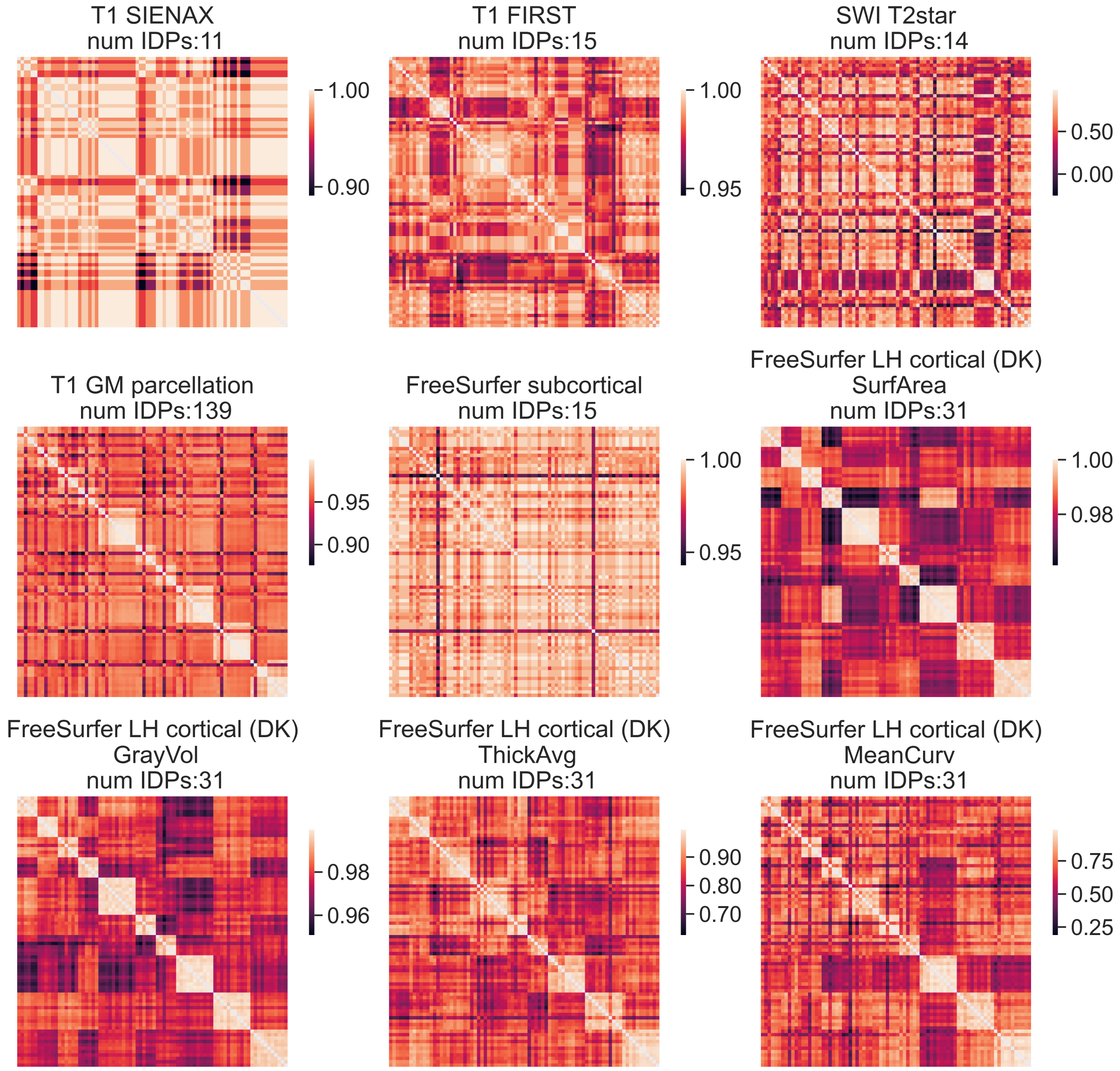

***Supplementary Figure 5a** – Correlation (Spearman's rank) matrices R_{ij}^{m} depicting the similarity of IDP categories (M_{cat}) between scanning sessions for all session pairs (i, j). The number of IDPs in each IDP category is also reported. IDP categories include subcortical volumes, brain tissue volumes, subcortical T2*, cortical parcel volumes, dMRI regional and tract-wise microstructure (FA, MD, MO, L1, L2, L3), rfMRI functional connectivity node amplitude and edges. For ease of visualisation, we show split the IDP categories to anatomical and dMRI/fMRI (following page).*

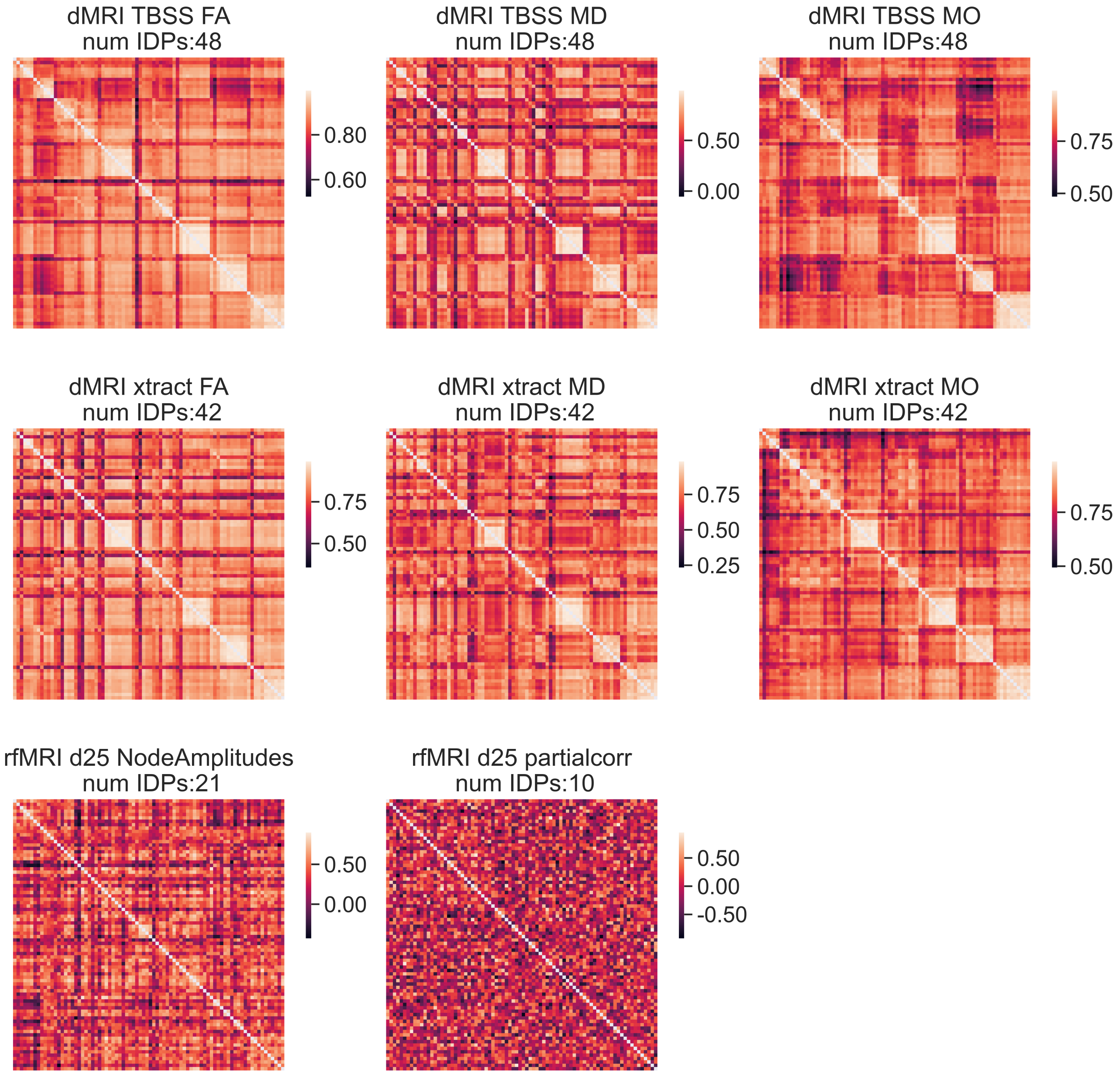

Supplementary Figure 5b *– As in Fig S4a but for dMRI and fMRI IDP categories including dMRI regional and tract-wise microstructure (FA, MD, MO, L1, L2, L3), rfMRI functional connectivity node amplitude and edges.*

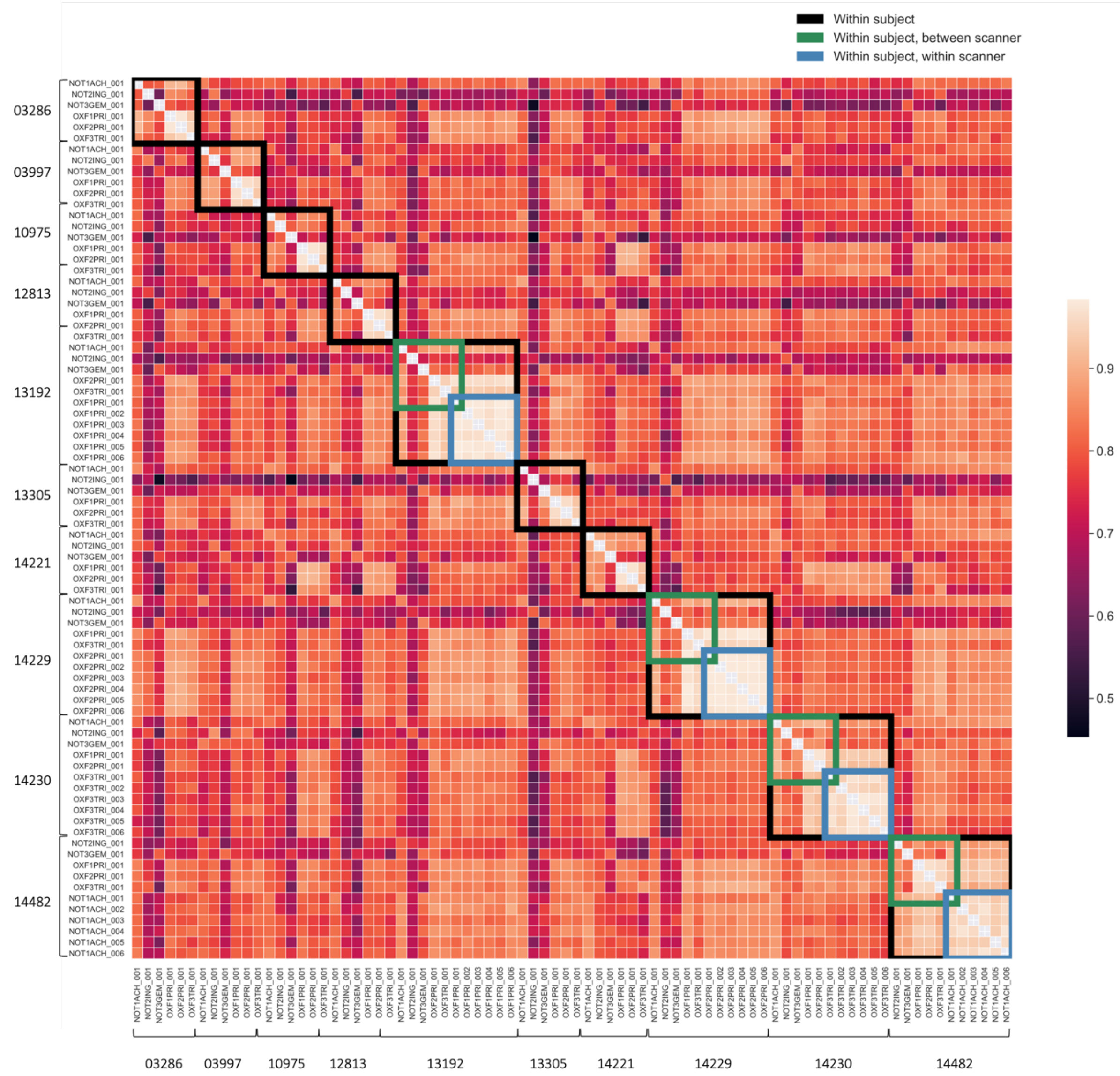

***Supplementary Figure 6** – The correlation (Spearman's rank) matrix P_{ij} depicting the median similarity of IDPs between scanning sessions for all sessions (i, j), calculated using Eq. 2.1 (Methods). Spearman's rank correlation is calculated for IDP categories (M_{cat}, presented in Supplementary Figure 4) and the median across categories plotted. Within-subject sessions are outlined in black. Within-scanner repeat sessions are outlined in blue. Between-scanner repeats are outlined in green.*

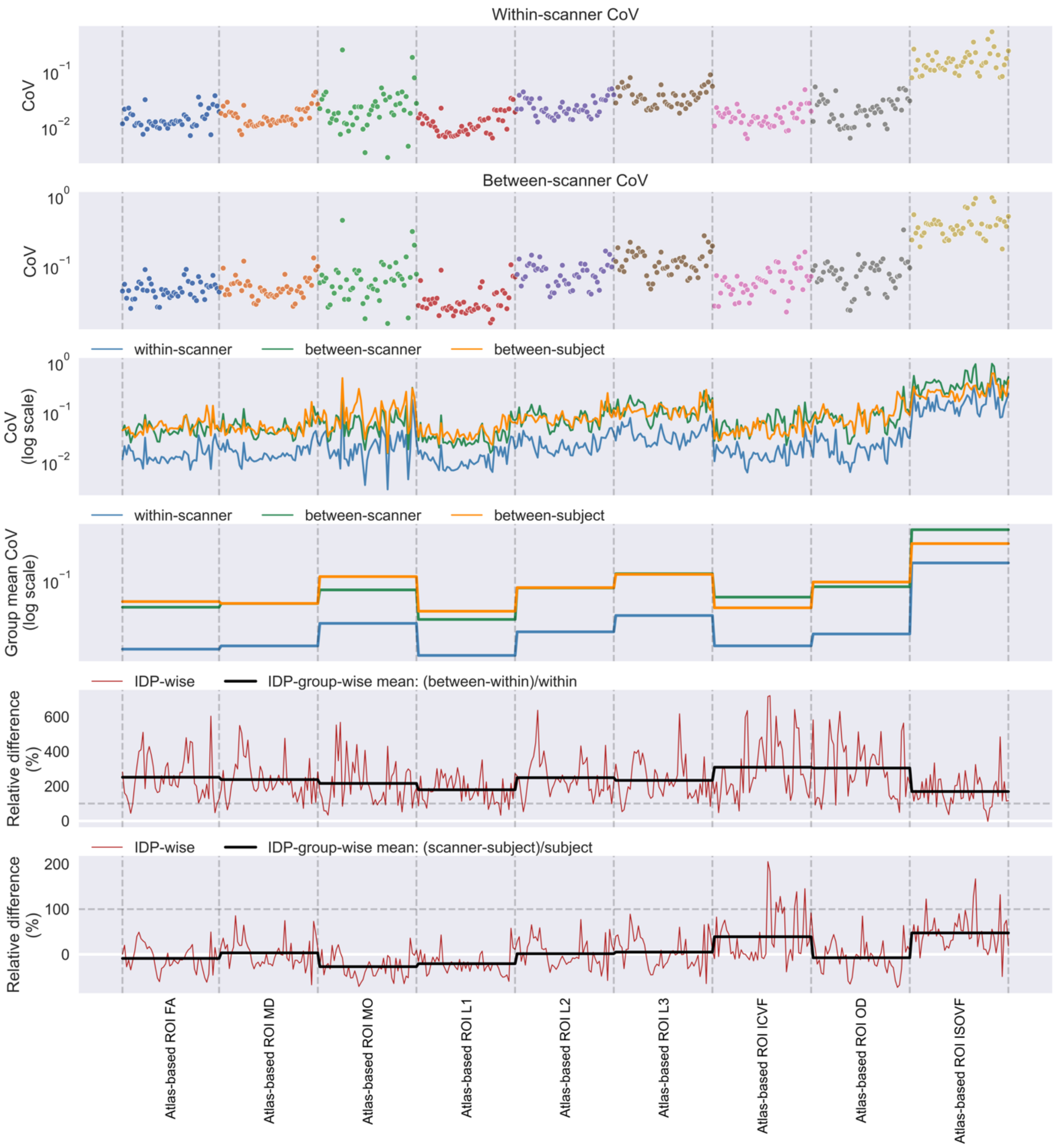

***Supplementary Figure 7** – The coefficient of variation of IDPs within/between scanner repeats for the atlas-based region wise diffusion metrics, including NODDI features. Top row: the IDP-wise CoVs across six within-scanner repeats, averaged across the four subjects with within-scanner repeats. Second row: the IDP-wise CoVs across six between-scanner repeats, averaged across all subjects. Third row: the within-scanner (blue), between-scanner (green) and between-subject-within-scanner (orange, reflecting biological variability) CoVs plotted on a log-scale. Fourth row: the IDP-group-wise mean of the CoVs (from the third row) plotted on a log scale for within-scanner (blue), between-scanner (green) and between-subject-within-scanner (orange) sessions. Fifth row: the IDP-wise (red) and IDP-group-wise (black) relative difference (between-within/within [scanner]) in CoVs. Bottom row: the IDP-group-wise relative difference in between-scanner CoVs (within scanner, blue; between-scanner, green) and between-subject (biological) CoVs. The dashed horizontal line in rows five and six indicate relative difference of 100%.*

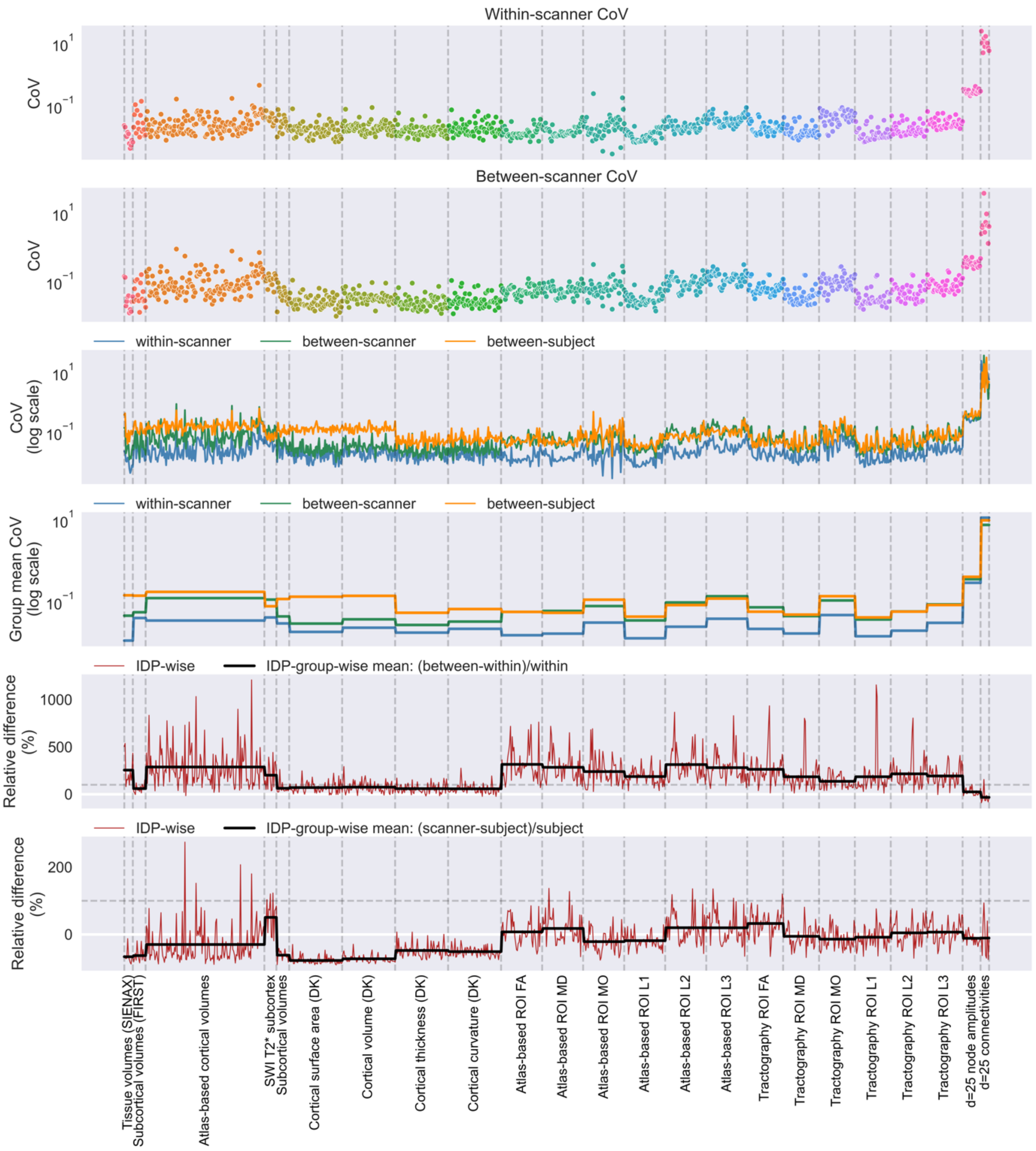

Supplementary Figure 8 *– As in Figure 6 but using only the four subjects with within-scanner repeats to calculate the within-scanner and between-scanner CoV. Top row: the IDP-wise CoVs across six within-scanner repeats, averaged across the four subjects with within-scanner repeats. Second row: the IDP-wise CoVs across six between-scanner repeats, averaged across the same four subjects. Third row: the within-scanner (blue), between-scanner (green) and between-subject-within-scanner (orange, reflecting biological variability) CoVs plotted on a log-scale. Fourth row: the IDP-group-wise mean of the CoVs plotted on a log scale for within-scanner (blue), between-scanner (green) and between-subject-within-scanner (orange) sessions. Fifth row: the IDP-wise (red) and IDP-group-wise (black) relative difference (between-within/within [scanner]) in CoVs. Bottom row: the IDP-group-wise relative difference between the scanner CoVs (within scanner, blue; between-scanner, green) and between-subject (biological) CoVs. The dashed horizontal line in rows five and six indicate relative difference of 100%.*

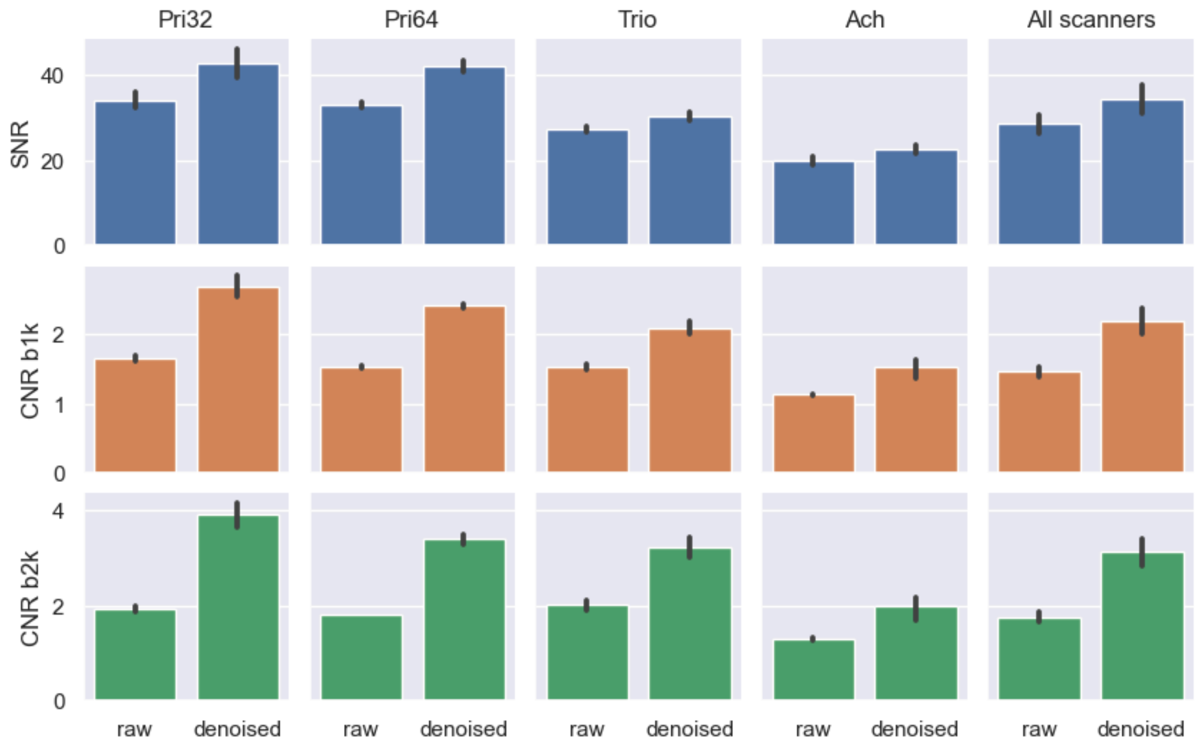

Supplementary Figure 9 *– Effect of diffusion MRI denoising on image quality metrics, including signal-to-noise ratio (SNR) and angular contrast-to-noise ratio (CNR) for different b values. Bars represent the mean (and standard deviation – error bars) of the reported IQM across subjects. Pri32: Siemens Prisma 32ch; Pri64: Siemens Prisma 64ch; Trio: Siemens Trio; Ach; Philips Achieva; All scanners: mean across scanners. SNR is reported for the b=0 s/mm^2 data; CNR b1k is the CNR for the b=1000 s/mm^2 data; CNR b2k is the CNR for the b=2000 s/mm^2 data.*

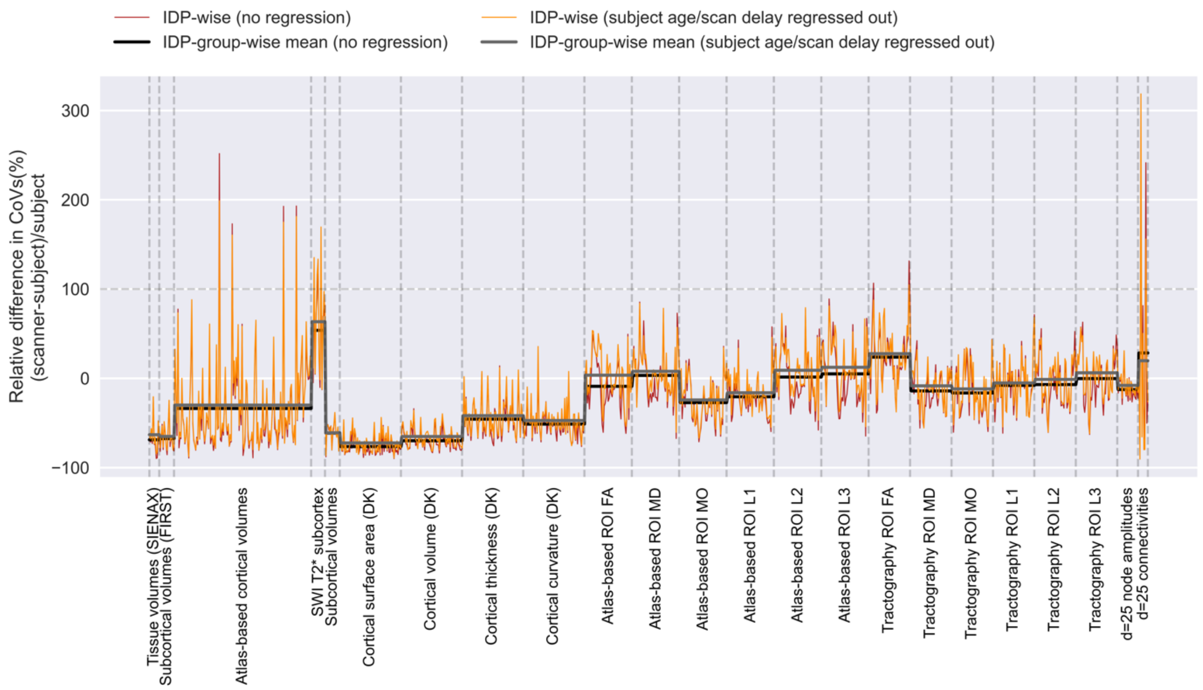

Supplementary Figure 10 – The effect of subject age and inter-scan delays on between-scanner and between-subject IDP variability. We repeated the analysis presented in Figure 6 (bottom plot) after regressing-out subject age and scan delays of the IDPs. We plot the IDP-wise (orange) and IDP-group-wise (grey) relative difference (between-scanner – between-subject/between-subject) in CoVs for the new (residualised) IDPs, and the original IDP-wise and IDP-group-wise mean data from Figure 6 (red and black lines respectively).
